# Type 2 diabetes has a protective causal association with thoracic aortic aneurysm: a Mendelian randomization study

**DOI:** 10.1186/s13098-023-01101-1

**Published:** 2023-06-07

**Authors:** Yiran Zhang, Yongxin Li, Xiaoyi Dai, Haokai Lin, Liang Ma

**Affiliations:** 1 Department of Cardiovascular Surgery, First Affiliated Hospital, School of Medicine, Zhejiang University, Hangzhou, 310003 China; 2 School of Public Health, Hangzhou Medical College, Hangzhou, Zhejiang China

**Keywords:** Type 2 diabetes, Glycated hemoglobin, Fasting glucose, Fasting insulin, Thoracic aortic aneurysm, Aortic diameter, Mendelian randomization

## Abstract

**Background:**

Observational studies have reported an inverse association of type 2 diabetes (T2D) with thoracic aortic aneurysm (TAA). However, the causality of the association has not been established yet. The present study aims to clarify the causal relationship between T2D and TAA via a Mendelian randomization (MR) approach.

**Methods:**

Causality of associations were assessed using a two-sample MR framework. Genome-wide association study (GWAS) summary statistics were obtained for T2D, glycated hemoglobin (HbA1c), fasting glucose (FG) and fasting insulin (FI) as exposures, and TAA, ascending aortic diameter (AAoD) and descending aortic diameter (DAoD) as outcomes. Four different methods (inverse variance weighted [IVW], weight median, MR-Egger and MR-PRESSO) were used to calculate causal estimates. Heterogeneity and horizontal pleiotropy were assessed using Cochran Q test and MR-Egger regression intercept, respectively.

**Results:**

Genetically predicted T2D was inversely associated with the risk of TAA (OR: 0.931, 95% CI 0.870 to 0.997, p = 0.040, IVW method) and AAoD (Beta: -0.065, 95%CI −0.099 to  − 0.031, p = 1.7e−04, IVW method), but not with DAoD (p > 0.05). Genetically predicted FG level was inversely associated with AAoD (Beta: −0.273, 95% CI −0.396 to –0.150, p = 1.41e−05, IVW method) and DAoD (Beta: −0.166, 95% CI −0.281 to −0.051, p = 0.005, IVW method), but not with TAA (p > 0.05). The effect of genetically predicted HbA1c and FI on TAA, AAoD and DAoD did not reach statistical significance (p > 0.05).

**Conclusions:**

Genetic predisposition to T2D decreases the risk of TAA. Genetically predicted T2D is inversely associated with AAoD, but not with DAoD. Genetically predicted FG level was inversely associated with AAoD and DAoD.

**Supplementary Information:**

The online version contains supplementary material available at 10.1186/s13098-023-01101-1.

## Background

Thoracic aortic aneurysm (TAA) is an insidious condition involving a progressive dilatation of thoracic aorta, with an incidence rate of 10.4 per 100,000 person-years [1]. TAA is usually asymptomatic until the presentation of fatal events such as aortic dissection or rupture, and the annual risk of sudden death from TAA due to acute aortic dissection is more than 10% [2]. Most of the TAAs are degenerative and develop in patients with risk factors for atherosclerosis such as hypertension, hyperlipidemia and smoking, while other risk factors include genetic predisposition, inflammation and infection [3].

Diabetes is an established risk factor for peripheral, coronary and cerebrovascular disease. However, numerous epidemiological studies have reported an inverse association of type 2 diabetes (T2D) with both TAA [4–6] and abdominal aortic aneurysm (AAA) [7, 8]. Various potential protective mechanisms have been proposed to mediate the inverse association between diabetes and aortic aneurysm, such as reduced aortic wall stress, reduced matrix metalloproteinases (MMPs) activity, increased glycation of extracellular matrix (ECM), regulation of inflammation, reduced aortic mural neoangiogenesis, maintaining the homeostasis of vascular smooth muscle cells (VSMCs), and protective effects from anti-diabetic therapy [9, 10]. However, it remains unclear whether the association between diabetes and aortic aneurysms are causal or may have arisen due to confounding bias.

Mendelian randomization (MR) is an epidemiological method using genetic variants associated with the exposure as instrumental variables (IVs) in non-experimental design to assess the causal effect of the exposure on the outcome [11]. MR takes advantage of the naturally occurring random allocation of alleles during the formation of the zygote, which can reduce both conventional confounding variables and reverse causation, providing better evidence of causal inference [12]. Several previous MR studies have shown that genetic predisposition to T2D did not play a causal role in AAA, which differed from tradition epidemiological evidences [13, 14]. There are distinctive features contrasting TAA and AAA, and it is suggested that TAA has stronger genetic components than AAA [9]. The causal relationship between T2D and TAA remains unclear.

The primary aim of our present study was to clarify the causal relationship between T2D and TAA via a Mendelian randomization approach. In addition, the effects of T2D on ascending aortic diameter (AAoD) and descending aortic diameter (DAoD) were also investigated to validate the primary result, and the effects of glycated hemoglobin (HbA1c), fasting glucose (FG) and fasting insulin (FI) on TAA/AAoD/DAoD were also investigated to explore the effect of hyperglycemia and insulin resistance on thoracic aortic disease.

## Material and methods

### Study design

**Fig. 1:**
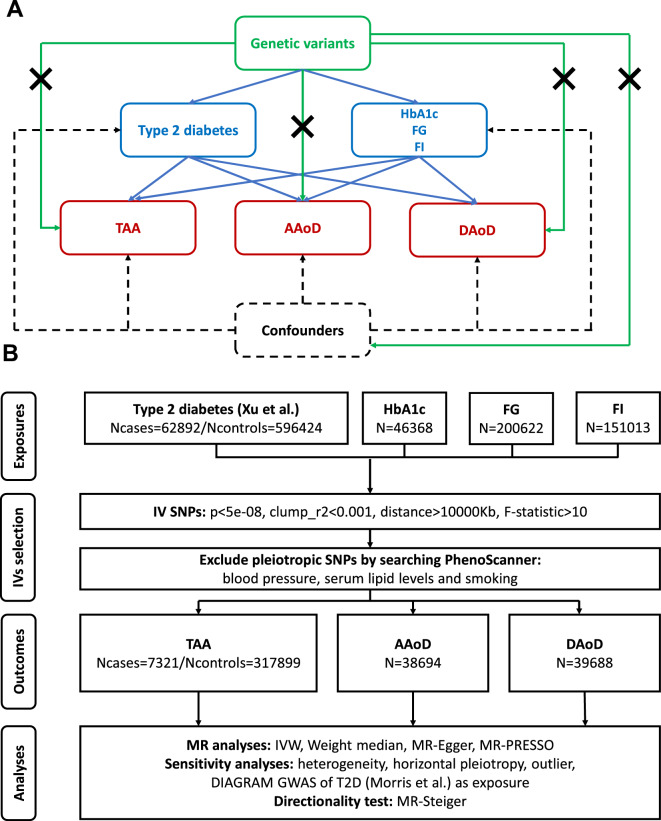
Study design of the present study. (**A**) Schematic representation of this MR study. (**B**) Flow diagram of the present MR framework. *TAA* thoracic aortic aneurysm, *AAoD* ascending aortic diameter, *DAoD* descending aortic diameter, *HbA1c* glycated hemoglobin, *FG* fasting glucose, *FI* fasting insulin, *IV* instrumental variable, *SNP* single nucleotide polymorphism, *MR* Mendelian randomization, *IVW* inverse variance weighted

The present two-sample MR study was conducted using summary data from published studies and publicly accessible resources to assess the causal relationship between T2D/HbA1c/FG/FI and TAA/AAoD/DAoD (Fig. 1A). The ethics committee at each institutional review board authorized all participants’ written informed permission in separate studies. No extra ethical approval or informed consent was required. Details about the data source, genetic instruments selection and statistical analyses were described as follows.

### GWAS datasets for exposures

The genome-wide association study (GWAS) summary data of T2D was from a large-scale meta-analysis conducted by Xu et al. combining 3 GWAS datasets (Genetics Replication and Meta-analysis [DIAGRAM], Genetic Epidemiology Research on Aging [GERA] and UK Biobank [UKB]), which contained a total of 62892 T2D cases and 596424 controls [15]. The GWAS summary data of HbA1c levels was from a large-scale meta-analysis containing a total of 46368 nondiabetic adults [16]. The participants in this study were free of diabetes as assessed by either clinical diagnosis, self-reported diabetes, diabetes treatment, or undiagnosed diabetes defined by fasting blood glucose≥7.0 mmol/l. HbA1c (in percentages) was measured from whole blood [16]. The GWAS summary data of FG and FI levels was from Meta-Analysis of Glucose and Insulin-related Traits Consortium (MAGIC) containing a total of 200622 (FG)/ 151013 (FI) nondiabetic adults [17]. Individuals in this study were excluded if they had diabetes diagnosed by a physician, reported use of diabetes-relevant medications; or had a FG≥7mmol/L, 2h glucose after an oral glucose challenge≥11.1mmol/L or HbA1c≥6.5%. FG (in mmol/L) and FI (in pmol/L) were measured from whole blood. Since the UKB samples in Xu’s T2D GWAS meta-analysis overlapped with the samples in outcome GWAS of AAoD and DAoD, we further utilized the DIAGRAM GWAS of T2D provided by Morris et al. (12171 T2D cases and 56862 controls) [18] to perform the sensitivity analyses. Details regarding study design, diagnostic criteria, participants selection, quality control and statistical analyses had been reported in the original studies. All the above populations are of European ancestry to minimize demographic heterogeneity. The GWAS summary data of T2D (GWAS ID: ebi-a-GCST006867 [Xu et al.], ieu-a-26 [Morris et al.]), HbA1c (GWAS ID: ieu-b-103), FG (GWAS ID: ebi-a-GCST90002232) and FI (GWAS ID: ebi-a-GCST90002238) were obtained from the IEU open GWAS project (https://gwas.mrcieu.ac.uk/). The characteristics of GWAS datasets for exposures were summarized in Table 1.

**Table 1:** Characteristics of GWAS datasets in this study

Contribution	Traits	Sample size	Population	References
Exposures	T2D	Cases: 62892/Controls: 596424	European	Xue et al
	T2D	Cases: 12171/Controls: 56862	European	Morris et al
	HbA1c	46368	European	Soranzo et al
	FG	200622	European	Chen et al
	FI	151013	European	Chen et al
Outcomes	TAA	Cases: 7321/Controls: 317899	European	FinnGen r8
	AAoD	38694	European	Pirruccello et al
	DAoD	39688	European	Pirruccello et al

*T2D* type 2 diabetes, *HbA1c* glycated hemoglobin, *FG* fasting glucose, *FI* fasting insulin, *TAA* thoracic aortic aneurysm, *AAoD* ascending aortic diameter, *DAoD* descending aortic diameter

### GWAS datasets for outcomes

The GWAS summary data of TAA was from the FinnGen Release 8 (https://www.finngen.fi/en), which contains 7321 TAA cases and 317899 controls from Finnish biobank. The diagnostic criteria for TAA are described in the following link (https://www.finngen.fi/en/researchers/clinical-endpoints). Details about the analytical process of GWAS data are described in the following link (https://finngen.gitbook.io/documentation/v/r8/). The GWAS summary data of aortic diameter was available in Cardiovascular Disease Knowledge Portal (CVDKP, https://cvd.hugeamp.org/). This GWAS dataset contains 38694 White British participants aged between 40 and 69 years in UK Biobank measured for AAoD (in millimeter) and 39688 measured for DAoD (in millimeter), using cardiac magnetic resonance images based on a deep learning model [19]. The characteristics of GWAS datasets for outcomes were summarized in Table 1.

### Selection of instrumental variables

MR studies utilize genetic variants closely related to the exposure as instrumental variables (IVs) to make causal inference of the exposure on the outcome outcome [11]. A genetic variant should meet 3 key assumptions to be selected as IV: (1) the IV should be directly associated with the exposure (relevance); (2) the IV should be independent of the confounding factors in the exposure-outcome association (independence); (3) the IV should not have a direct association with the outcome (exclusion) [11]. We set p < 5e−08 as the genome-wide significant threshold to select genetic variants strongly related to the outcomes. Then these genetic variants were clumped at a threshold of linkage disequilibrium (r^2^ = 0.001, distance = 10000 Kb). Besides, the instrument strength was estimated using F-statistic, and weak instruments with F-statistic<10 were removed [20]. To avoid potential pleiotropic effect, PhenoScanner (http://www.phenoscanner.medschl.cam.ac.uk) was utilized to scan each IV, and IVs associated with known risk factors of TAA including blood pressure, serum lipid levels and smoking in European population were removed (using p = 1e−05 and R^2^ = 0.8 as thresholds; Additional file 1: Table S8–S12).

### Mendelian randomization

The selected IVs were extracted from the outcome traits and were harmonized in both exposure and outcome GWAS. If a particular requested single nucleotide polymorphism (SNP) was not present in the outcome GWAS, a proxy SNP that is in linkage disequilibrium with the target SNP would be searched using 1000 genomes European sample data (R^2^>0.8). Palindromic SNPs and ambiguous SNPs were discarded. Four different methods (inverse variance weighted, weight median, MR-Egger and MR-PRESSO) were used to perform two-sample MR, and we considered the association as causal when at least three methods provided consistent results. Inverse variance weighted (IVW) method was chosen as the main MR analysis since it is the most efficient analysis with valid IVs [21]. The Cochran Q test for IVW was used to measure heterogeneity. When the p value of Cochran Q test > 0.05, which suggested significantly heterogeneous, IVW with random effects was performed. Weighted median method can generate credible estimates when more than 50% of the weight comes from valid IVs [22]. MR-Egger method was used as the main estimation to account for potential pleiotropy [23]. MR-PRESSO method was used to correct possible horizontal pleiotropic outlier IVs [24]. MR-Egger regression intercept was used to test for horizontal pleiotropy (p<0.05 was judged significant) [23]. Direction of effect was assessed using MR-Steiger [25]. TwoSampleMR (version 0.5.6), MR-PRESSO (version 1.0), and phenoscanner (version 1.0) packages in R (version 4.2.2) were used to conduct the analysis. The present study was not pre-registered at any platform. The flowchart of the present study was presented in Fig. 1B.

## Results

### Effects of T2D on TAA, AAoD and DAoD

**Fig. 2:**
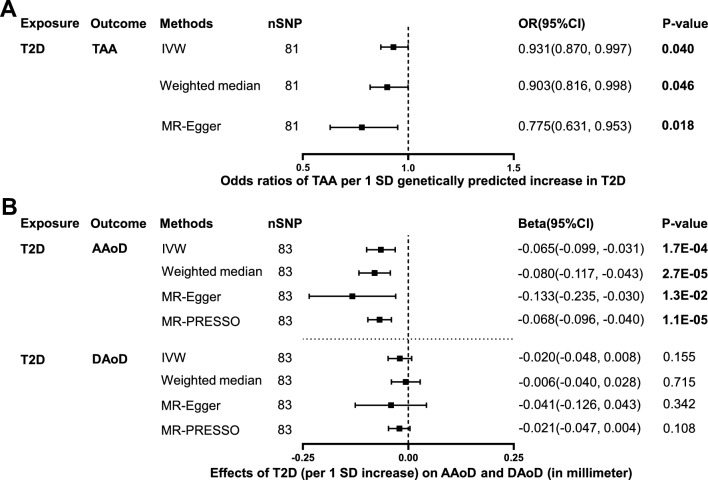
Effect of genetically predicted T2D on TAA, AAoD and DAoD. Forest plot of MR estimated the effect of T2D on risk of TAA (**A**) and the effect of T2D on AAoD and DAoD (**B**). *T2D* type 2 diabetes, *TAA* thoracic aortic aneurysm, *AAoD* ascending aortic diameter, *DAoD* descending aortic diameter, *IVW* inverse variance weighted

**Fig. 3:**
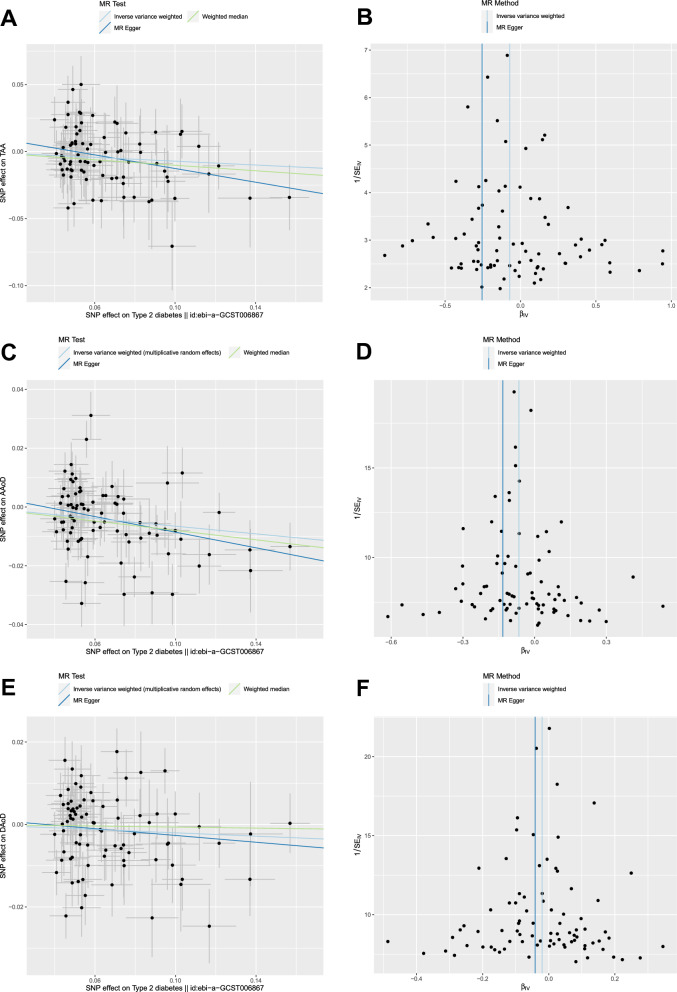
Scatter plots and funnel plots of MR analyses for T2D with TAA, AAoD and DAoD. Scatter plot (**A**) and funnel plot (**B**) of MR analysis for T2D with TAA. Scatter plot (**C**) and funnel plot (**D**) of MR analysis for T2D with AAoD. Scatter plot (**E**) and funnel plot (**F**) of MR analysis for T2D with DAoD. *T2D* type 2 diabetes, *TAA* thoracic aortic aneurysm, *AAoD* ascending aortic diameter, *DAoD* descending aortic diameter

81 independent SNPs associated with T2D were selected as IVs to estimate the effect of T2D on TAA (Additional file 1: Table S1). The F-statistics of these IVs were all greater than 10 (ranging from 29.9 to 256.3), indicating a low risk of weak instrument bias. Genetically predicted T2D (per 1 SD increase) was inversely associated with the risk of TAA using IVW method (odds ratio [OR]: 0.931, 95% confidence interval [CI]: 0.870 to 0.997, p = 0.040; Fig. 2 and Additional file 1: Table S3). In addition, weighted median method (OR: 0.903, 95%CI 0.816 to 0.998, p = 0.046) and MR-Egger method (OR 0.775, 95% CI 0.631 to 0.953, p = 0.018) showed consistent results (Fig. 2 and Additional file 1: Table S3). No significant heterogeneity was present according to the Cochran Q test (Q = 84.9, p = 0.332; Additional file 1: Table S2). No significant horizontal pleiotropy was observed according to the MR-Egger regression intercept test (intercept = 0.013, p = 0.069; Additional file 1: Table S2). The effective value of individual IVs of T2D on TAA was demonstrated by scatter plot (Fig. 3A), funnel plot (Fig. 3B) and forest plot (Additional file 3: Figure S2A). The causal assumption of T2D and TAA was verified through the MR-Steiger test, which validated that the effect of T2D on TAA was the correct causal direction (p = 5.35e−15).

83 independent SNPs associated with T2D were selected as IVs to estimate the effect of T2D on AAoD and DAoD (Additional file 1: Table S1). Genetically predicted T2D (per 1 SD increase) was inversely associated with AAoD (in millimeter) using IVW method (Beta: −0.065, 95% CI −0.099 to −0.031, p = 1.7e−04; Fig. 2 and Additional file 1: Table S4). Weighted median method (Beta: -0.080, 95% CI −0.117 to −0.043, p = 2.7e−05), MR-Egger method (Beta: −0.133, 95% CI −0.235 to −0.030, p = 0.013), and MR-PRESSO method (Beta: −0.068, 95% CI −0.096 to −0.040, p = 1.1e−05; outlier IVs corrected) showed consistent results (Fig. 2 and Additional file 1: Table S4). Cochran Q test showed significant heterogeneity (Q = 174.9, p = 1.10e−08; Additional file 1: Table S2), while no significant horizontal pleiotropy was observed (intercept = 0.005, p = 0.176; Additional file 1: Table S2). The effective value of individual IVs of T2D on AAoD was demonstrated by scatter plot (Fig. 3C), funnel plot (Fig. 3D) and forest plot (Additional file 3: Figure S2B). The causal assumption of T2D and AAoD was verified through the MR-Steiger test, which validated that the effect of T2D on AAoD was the correct causal direction (p = 2.22e−76).

The effect of genetically predicted T2D (per 1 SD increase) on DAoD (in millimeter) did not reach statistical significance in all 4 MR methods (p > 0.05, Fig. 2 and Additional file 1: Table S4). Cochran Q test showed significant heterogeneity (Q = 148.5, p = 9.84e−06; Additional file 1: Table S2), while no significant horizontal pleiotropy was observed (intercept = 0.001, p = 0.607; Additional file 1: Table S2). The effective value of individual IVs of T2D on DAoD was demonstrated by scatter plot (Fig. 3E), funnel plot (Fig. 3F) and forest plot (Additional file 3: Figure S2C).

The sensitivity analyses using the DIAGRAM GWAS of T2D showed similar results with the primary analyses. 7 independent SNPs associated with T2D were selected as IVs to estimate the effect of T2D on AAoD and DAoD (Additional file 1: Table S5). Genetically predicted T2D (per 1 SD increase) was inversely associated with AAoD (in millimeter) using IVW method (Beta: −0.057, 95% CI −0.095 to −0.019, p = 0.003; Additional file 1: Table S7) and Weighted median method (Beta: −0.055, 95% CI −0.103 to −0.007, p = 0.024; Additional file 1: Table S7), while the result of MR-Egger method was not significant (Beta: -−0.098, 95% CI −0.355 to 0.158, p = 0.487; Additional file 1: Table S7). The effect of genetically predicted T2D on DAoD did not reach statistical significance (p > 0.05, Additional file 1: Table S7). No significant heterogeneity or horizontal pleiotropy were observed (Additional file 1: Table S6).

### Effects of HbA1c on TAA, AAoD and DAoD

**Fig. 4:**
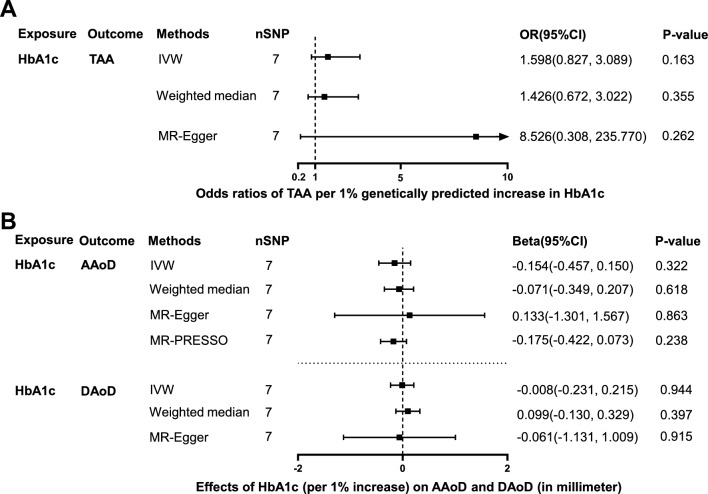
Effect of genetically predicted HbA1c on TAA, AAoD and DAoD. Forest plot of MR estimated the effect of HbA1c level on risk of TAA (**A**) and the effect of HbA1c level on AAoD and DAoD (**B**). *HbA1c* glycated hemoglobin, *TAA* thoracic aortic aneurysm, *AAoD* ascending aortic diameter, *DAoD* descending aortic diameter, *IVW* inverse variance weighted

7 independent SNPs associated with HbA1c were selected as IVs to estimate the effect of HbA1c on TAA, AAoD and DAoD (Additional file 1: Table S1). The F-statistics of these IVs were all greater than 10 (ranging from 32.8–56.7), indicating a low risk of weak instrument bias. The effect of genetically predicted HbA1c (per 1% increase) on TAA, AAoD and DAoD did not reach statistical significance in all 4 MR methods (p > 0.05, Fig. 4, Additional file 1: Table S3 and Table S4). No significant horizontal pleiotropy was observed in the associations of HbA1c with TAA, AAoD and DAoD (p > 0.05, Additional file 1: Table S2). Cochran Q test showed significant heterogeneity in the association between HbA1c and AAoD (Q = 17.3, p = 0.008; Additional file 1: Table S2), while no significant heterogeneity was present in the associations of HbA1c with TAA and DAoD (p > 0.05; Additional file 1: Table S2). The scatter plots and funnel plots were presented in Additional file 2: Figure S1, and the forest plots were presented in Additional file 3: Figure S2D-F.

### Effects of FG on TAA, AAoD and DaoD

**Fig. 5:**
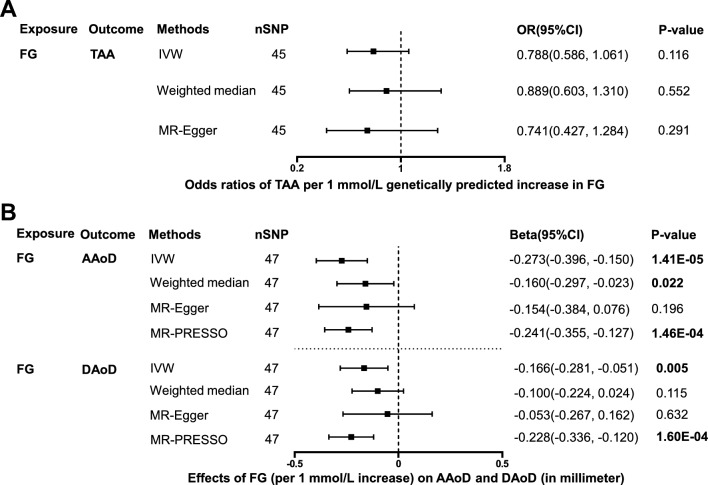
Effect of genetically predicted FG on TAA, AAoD and DAoD. Forest plot of MR estimated the effect of FG level on risk of TAA (**A**) and the effect of FG level on AAoD and DAoD (**B**). *FG* fasting glucose, *TAA* thoracic aortic aneurysm, *AAoD* ascending aortic diameter, *DAoD* descending aortic diameter, *IVW* inverse variance weighted

45 independent SNPs associated with FG were selected as IVs to estimate the effect of FG on TAA (Additional file 1: Table S1). The effect of genetically predicted FG level (per 1 mmol/L increase) on TAA did not reach statistical significance in all 3 MR methods (p > 0.05, Fig. 5, Additional file 1: Table S3). No significant heterogeneity was present according to the Cochran Q test (Q = 58.8, p = 0.067; Additional file 1: Table S2). No significant horizontal pleiotropy was observed according to the MR-Egger regression intercept test (intercept = 0.002, p = 0.793; Additional file 1: Table S2). The scatter plots and funnel plots were presented in Additional file 4: Figure S3A-B, and the forest plots were presented in Additional file 3: Figure S2G.

47 independent SNPs associated with FG were selected as IVs to estimate the effect of FG on AAoD and DAoD (Additional file 1: Table S1). Genetically predicted FG level (per 1 mmol/L increase) was inversely associated with AAoD (in millimeter) using IVW method (Beta: −0.273, 95% C: −0.396 to −0.150, p = 1.41e−05), Weighted median method (Beta: −0.160, 95% CI −0.297 to −0.023, p = 0.022) and MR-PRESSO method (Beta: −0.241, 95% CI −0.355 to −0.127, p = 1.46e−04; outlier IVs corrected), while the result using MR-Egger method was not significant (Beta: −0.154, 95% CI −0.384 to 0.076, p = 0.196; Fig. 5 and Additional file 1: Table S4). Cochran Q test showed significant heterogeneity (Q = 82.4, p = 7.80e−04; Additional file 1: Table S2), while no significant horizontal pleiotropy was observed (intercept = −0.003, p = 0.237; Additional file 1: Table S2). The scatter plots and funnel plots were presented in Additional file 4: Figure S3C, D, and the forest plots were presented in Additional file 3: Figure S2H. The causal assumption of FG and AAoD was verified through the MR-Steiger test, which validated that the effect of FG on AAoD was the correct causal direction (p = 0.001).

Genetically predicted FG level (per 1 mmol/L increase) was inversely associated with DAoD (in millimeter) using IVW method (Beta: −0.166, 95% CI −0.281 to −0.051, p = 0.005) and MR-PRESSO method (Beta: −0.228, 95% CI −0.336 to −0.120, p = 1.60e−04; outlier IVs corrected), while the results using Weighted median method (Beta: −0.100, 95% CI −0.224 to 0.024, p = 0.115) and MR-Egger method (Beta: −0.053, 95% CI −0.267 to 0.162, p = 0.632) were not significant (Fig. 5 and Additional file 1: Table S4). Cochran Q test showed significant heterogeneity (Q = 91.6, p = 7.43e−05; Additional file 1: Table S2), while no significant horizontal pleiotropy was observed (intercept = −0.003, p = 0.228; Additional file 1: Table S2). The scatter plots and funnel plots were presented in Additional file 4: Figure S3E-F, and the forest plots were presented in Additional file 3: Figure S2I. The causal assumption of FG and DAoD was verified through the MR-Steiger test, which validated that the effect of FG on DAoD was the correct causal direction (p = 1.77e−04).

### Effects of FI on TAA, AAoD and DAoD

**Fig. 6:**
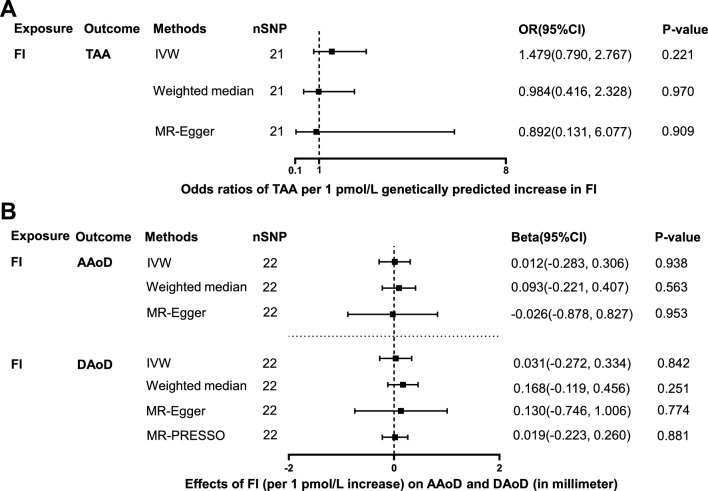
Effect of genetically predicted FI on TAA, AAoD and DAoD. Forest plot of MR estimated the effect of FI level on risk of TAA (**A**) and the effect of FI level on AAoD and DAoD (**B**). *FI* fasting insulin, *TAA* thoracic aortic aneurysm, *AAoD* ascending aortic diameter, *DAoD* descending aortic diameter, *IVW* inverse variance weighted

21 independent SNPs associated with FI were selected as IVs to estimate the effect of FI on TAA, and 22 independent SNPs associated with FI were selected as IVs to estimate the effect of FI on AAoD and DAoD (Additional file 1: Table S1). The effect of genetically predicted FI (per 1 pmol/L) on TAA, AAoD and DAoD did not reach statistical significance in all 4 MR methods (p > 0.05, Fig. 6, Additional file 1: Tables S3 and S4). No significant horizontal pleiotropy was observed in the associations of FI with TAA, AAoD and DAoD (p > 0.05, Additional file 1: Table S2). Cochran Q test showed significant heterogeneity in the association of FI with AAoD (Q = 48.5, p = 5.82e−04) and DAoD (Q = 65.4, p = 1.90e−06; Additional file 1: Table S2), while no significant heterogeneity was present in the associations of FI with TAA (p > 0.05; Additional file 1: Table S2). The scatter plots and funnel plots were presented in Additional file 5: Figure S4, and the forest plots were presented in Additional file 3: Figure S2J–L.

## Discussion

The present MR study showed that genetic predisposition to T2D was inversely associated with TAA and AAoD, but not with DAoD. Genetically predicted FG level was inversely associated with AAoD and DAoD, but not with TAA. In contrast, there was no evidence of causal association between genetically predicted HbA1c or FI levels and TAA, AAoD or DAoD.

Previous epidemiological studies have indicated an inverse association of T2D with TAA, while insufficient for ensuring a causal inference. A large nationwide case–control study using inpatient data in US showed that diabetes was significantly negatively associated with thoracic aortic aneurysm and dissection (TAAD) after adjusting for clinical risk factor differences (OR 0.48, 95% CI 0.44 to 0.52). Besides, compared to patients discharged without diabetes, those with chronic complications of diabetes were least likely to have TAAD (OR 0.17, 95% CI 0.12–0.23) [5]. A meta-analysis pooled the results from 5 case–control studies and 5 cohort studies, which demonstrated an inverse association between diabetes and TAA (OR 0.77, 95% CI 0.61 to 0.98) [6]. A large cross-sectional study of computed tomographic scans on 21295 patients also found that diabetes was associated with lower risk for ascending TAA (OR 0.60, 95% CI 0.40 to 0.87), using AAoD ≥ 4.5 cm as threshold [4]. The results of our present study support the previous observational studies that suggest T2D is a protective factor to TAA. Moreover, due to the nature of MR framework, which is less susceptible to residual environmental confounding factors than traditional observational studies, our MR study results indicated an unconfounded relationship between T2D and TAA, and suggested that this protective association is likely to be causal.

A previous wide-angled Mendelian randomization study investigating the effect of T2D liability on 12 cardiovascular diseases showed no significant association between T2D and TAA (Liu et al.; OR 0.973, 95% CI 0.859–1.102) [26]. It should be noted that there are several strengths of our present study. Firstly, our study used the FinnGen cohort as outcome dataset for TAA, containing more cases (7321 cases) compared with the UK Biobank cohort used in Liu et al.’s study (347 cases), which increased the statistical power. Secondly, our present study removed IVs associated with known risk factors of TAA including blood pressure, serum lipid levels and smoking (Additional file 1: Table S8–S12), which complied with the independence assumption of MR and reduced the horizontal pleiotropy. Moreover, our present study used aortic diameter GWAS as outcome datasets to further validate our primary results, which increased the robustness of our findings.

In order to explore the direct effect of hyperglycemia on thoracic aortic disease, we further investigated the effects of genetically predicted FG and HbA1c level on TAA, AAoD and DAoD. Interestingly, the results suggested that genetically predicted FG level was inversely associated with AAoD and DAoD. However, the effects of FG on TAA did not reach statistical significance. In contrast, there was no evidence of causal association between genetically predicted HbA1c level and TAA, AAoD or DAoD. These results were consistent with a recent cohort study in a large population of 3,358,293 individuals, which observed that a dose-dependent decrease in the risk of aortic aneurysm or dissection was associated with higher FG level. Briefly, hazard ratio of FG level per 10 mg/dl was 0.95 (95% CI 0.92–0.98) for aortic aneurysm [27]. HbA1c is not only determined by glycemia but is also affected by the rate of hemoglobin glycation, which depends on erythrocyte properties [28]. A previous MR study suggested that HbA1c underestimated fasting glucose in men compared with women, possibly due to erythrocyte properties [29].. These evidences might explain the inconsistent results between FG and HbA1c in the present MR study. Meanwhile, it should be noted that the effect of hyperglycemia on aortic aneurysm remained controversial. A previous study in a large population of 3,276,139 adults suggested an opposite association of aortic aneurysm with blood glucose and with diabetes. Briefly, that study showed that diabetes was associated with 22% lower prevalence of aortic aneurysm (OR 0.78, 95%CI 0.74–0.83), while in people without diabetes, higher usual blood glucose was significantly positively associated with a higher prevalence of aortic aneurysm (OR 1.22, 95% CI 1.04–1.43) [30]. Further mechanism studies are still needed to validate the effect of hyperglycemia on aortic disease.

Various studies have suggested the protective roles of different anti-diabetic medications against aortic aneurysm. Metformin is one of the first line oral hypoglycemic agents, which is the mostly commonly prescribed medication for diabetes worldwide. Several epidemiological studies have suggested that metformin prescription was associated with decreased AAA enlargement rate, surgical repair of AAA, or AAA related mortality, while no other diabetes treatment was found to be associated with AAA progression [31–34]. A study in patients with polycystic ovary syndrome showed that adding metformin to oral contraceptive pills treatment could improve the elastic parameters of the aorta [35]. In an experimental AAA model created by transient intra-aortic porcine pancreatic elastase infusion in normoglycemic mice, administration of metformin was found to suppress both AAA formation and progression, and was associated with aortic medial elastin and smooth muscle cells preservation, reduced immune cell infiltration, and reduced mural neovessel density [34]. Another study showed that metformin attenuated angiotensin II induced aortic aneurysm in ApoE(-/-) mice by reducing monocyte infiltration [36]. These evidences suggested that metformin might have pleiotropic anti-inflammatory and vascular protective effects on aorta.

The present MR study suggested that genetically predicted T2D was inversely associated with AAoD, while the effect of T2D on DAoD did not reach statistical significance. The GWAS of thoracic aortic diameters showed a limited locus overlap of the ascending and descending thoracic aorta, which highlighted their distinct genetic background. Besides, the polygenic score built from the ascending aorta GWAS showed a stronger association with thoracic aortic aneurysm or dissection than that from descending aorta GWAS[19]. A cross-sectional study assessing thoracic aorta calcification in T2D patients showed that higher calcium score of descending aorta, but not ascending aorta, was associated with peripheral arterial disease [37]. A study evaluating calcification score of coronary arteries and aorta suggested that ascending thoracic aneurysm and type A aortic dissection was associated with decreased systemic atherosclerosis [38]. Another study also found that patients with ascending aortic aneurysms had lower carotid intima-media thickness values [39]. These evidences suggest that it may be worth viewing ascending and descending aortic aneurysms as 2 separate phenotypes while studying the effect and underlying mechanisms of diabetes on aortic aneurysm.

There are several limitations of the present study. First, the study populations were of European ancestry, whether the findings of the present study were universal for other ethnic groups remains to be determined. Secondly, the association between HbA1c or FI levels and higher risk of TAA not achieving statistical significance cannot rule out potential causal effects, as the negative results in a MR study might be due to insufficient power of IVs or relatively small sample size. Moreover, although the present MR study provided evidences for the causal relationship between T2D and TAA, intervention experiments are still needed to clarify the functional mechanisms underlying the causal association.

## Conclusions

In conclusion, the present study is the first MR research to evaluate the causal relationship between T2D and TAA, which provides evidence supporting the protective causal effect of genetically predicted T2D on TAA. In addition, genetically predicted T2D was inversely associated with AAoD, but not with DAoD, while genetically predicted FG level was inversely associated with both AAoD and DAoD.

## Supplementary Information

**Additional file 1: Table S1.** Genetic instruments for T2D, HbA1c, FG and FI, with corresponding SNP effects for TAA, AAoD and DAoD used in the MR analysis. **Table S2.** Heterogeneity and horizontal pleiotropy tests of T2D/HbA1c/FG/FI on TAA/AAoD/DAoD. **Table S3.** Mendelian randomization results for effect of T2D/HbA1c/FG/FI on TAA. **Table S4.** Mendelian randomization results for effect of T2D/HbA1c/FG/FI on AAoD/DAoD. **Table S5.** Genetic instruments for T2D (Morris et al.), with corresponding SNP effects for AAoD and DAoD used in the MR analysis. **Table S6.** Heterogeneity and horizontal pleiotropy tests of T2D (Morris et al.) on AAoD/DAoD. **Table S7.** Mendelian randomization results for effect of T2D (Morris et al.) on AAoD/DAoD. **Table S8.** Removed SNPs for T2D (Xu et al.) (scaned using PhenoScanner). **Table S9.** Removed SNPs for HbA1c (scaned using PhenoScanner). **Table S10.** Removed SNPs for FG (scaned using PhenoScanner). **Table S11.** Removed SNPs for FI (scaned using PhenoScanner). **Table S12.** Removed SNPs for T2D (Morris et al.) (scaned using PhenoScanner).

**Additional file 2: Figure S1.** Scatter plots and funnel plots of MR analyses for HbA1c with TAA, AAoD and DAoD. Scatter plot (A) and funnel plot (B) of MR analysis for HbA1c with TAA. Scatter plot (C) and funnel plot (D) of MR analysis for HbA1c with AAoD. Scatter plot (E) and funnel plot (F) of MR analysis for HbA1c with DAoD. HbA1c, glycated hemoglobin; TAA, thoracic aortic aneurysm; AAoD, ascending aortic diameter; DAoD, descending aortic diameter.

**Additional file 3: Figure S2.** Forest plots of MR analyses for T2D with TAA (A), AAoD (B) and DAoD (C), for HbA1c with TAA (D), AAoD (E) and DAoD (F), for FG with TAA (G), AAoD (H) and DAoD (I), and for FI with TAA (J), AAoD (K) and DAoD (L). T2D, type 2 diabetes; HbA1c, glycated hemoglobin; FG, fasting glucose; FI, fasting insulin; TAA, thoracic aortic aneurysm; AAoD, ascending aortic diameter; DAoD, descending aortic diameter.

**Additional file 4: Figure S3.** Scatter plots and funnel plots of MR analyses for FG with TAA, AAoD and DAoD. Scatter plot (A) and funnel plot (B) of MR analysis for FG with TAA. Scatter plot (C) and funnel plot (D) of MR analysis for FG with AAoD. Scatter plot (E) and funnel plot (F) of MR analysis for FG with DAoD. FG, fasting glucose; TAA, thoracic aortic aneurysm; AAoD, ascending aortic diameter; DAoD, descending aortic diameter.

**Additional file 5: Figure S4.** Scatter plots and funnel plots of MR analyses for FI with TAA, AAoD and DAoD. Scatter plot (A) and funnel plot (B) of MR analysis for FI with TAA. Scatter plot (C) and funnel plot (D) of MR analysis for FI with AAoD. Scatter plot (E) and funnel plot (F) of MR analysis for FI with DAoD. FI, fasting insulin; TAA, thoracic aortic aneurysm; AAoD, ascending aortic diameter; DAoD, descending aortic diameter.

## Data Availability

Publicly available datasets were analyzed in this study. These data can be found here: IEU open GWAS project (https://gwas.mrcieu.ac.uk/), FinnGen Release 8 (https://www.finngen.fi/en), and Cardiovascular Disease Knowledge Portal (https://cvd.hugeamp.org/).

## Abbreviations

TAA: Thoracic aortic aneurysm
T2D: Type 2 diabetes
AAA: Abdominal aortic aneurysm
MMPs: Matrix metalloproteinases
ECM: Extracellular matrix
VSMCs: Vascular smooth muscle cells
MR: Mendelian randomization
IVs: Instrumental variables
AAoD: Ascending aortic diameter
DAoD: Descending aortic diameter
HbA1c: Glycated hemoglobin
FG: Fasting glucose
FI: Fasting insulin
GWAS: Genome-wide association study
SNP: Single nucleotide polymorphism
IVW: Inverse variance weighted
OR: Odds ratio
CI: Confidence interval
