# Supplementary figures and images for: Type 2 diabetes has a protective causal association with thoracic aortic aneurysm: a Mendelian randomization study
Source: Diabetol Metab Syndr. 2023 Jun 7;15:120. doi: 10.1186/s13098-023-01101-1 (PMC10245418; doi:10.1186/s13098-023-01101-1)

**A**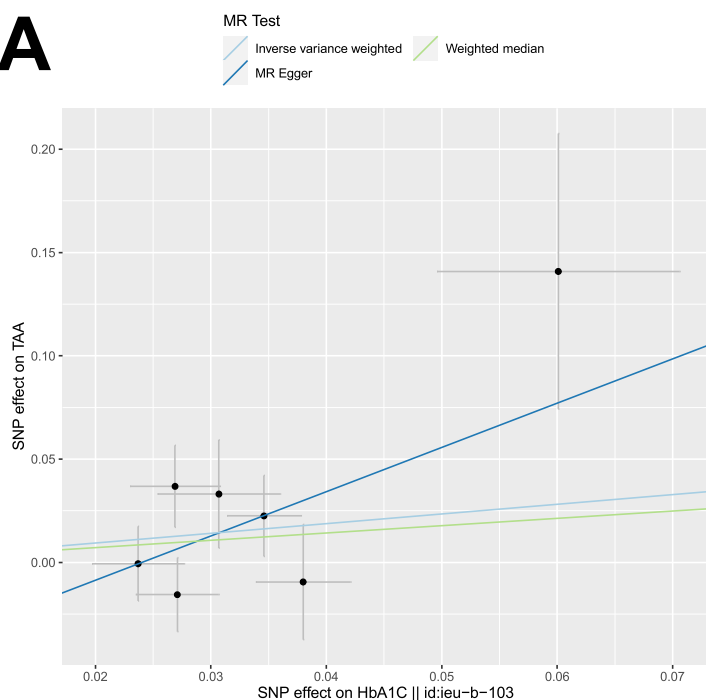**B**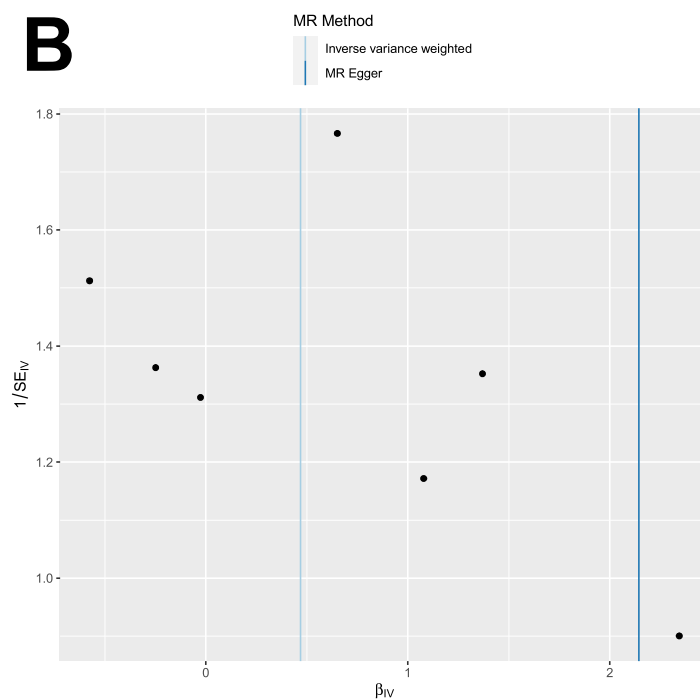**C**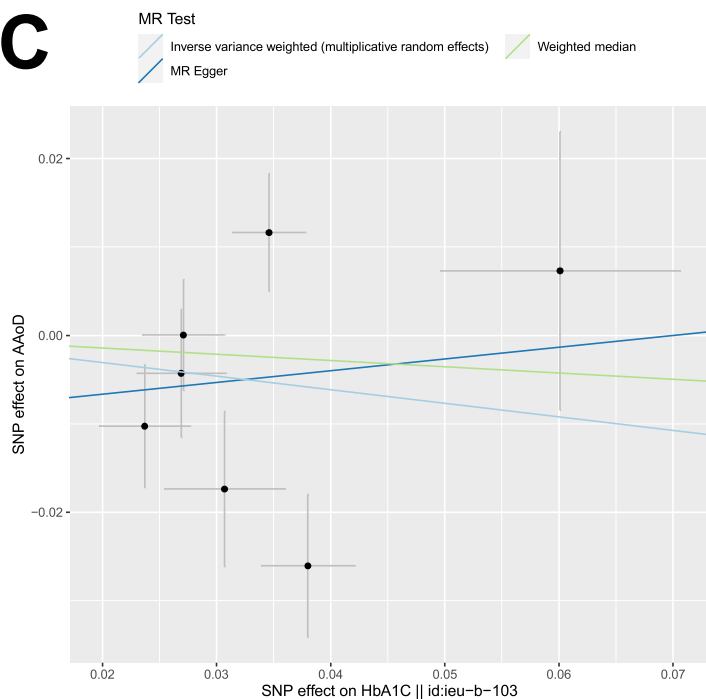**D**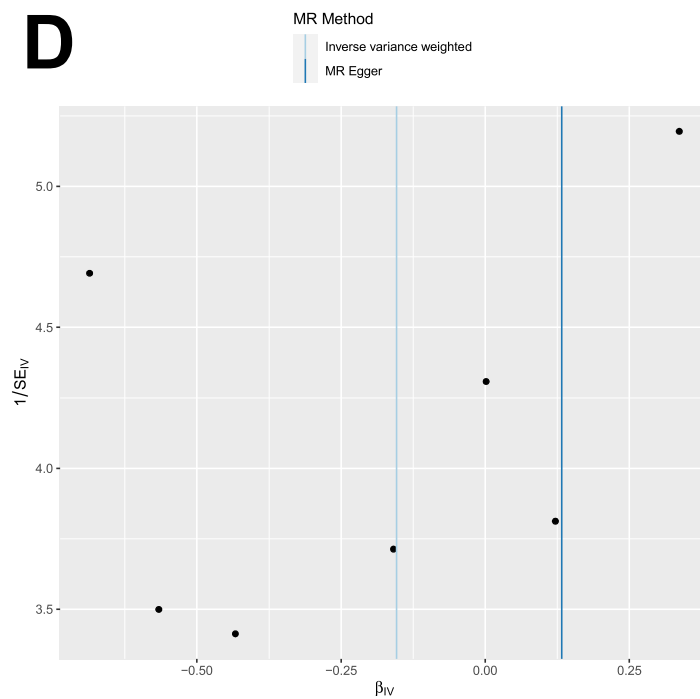**E**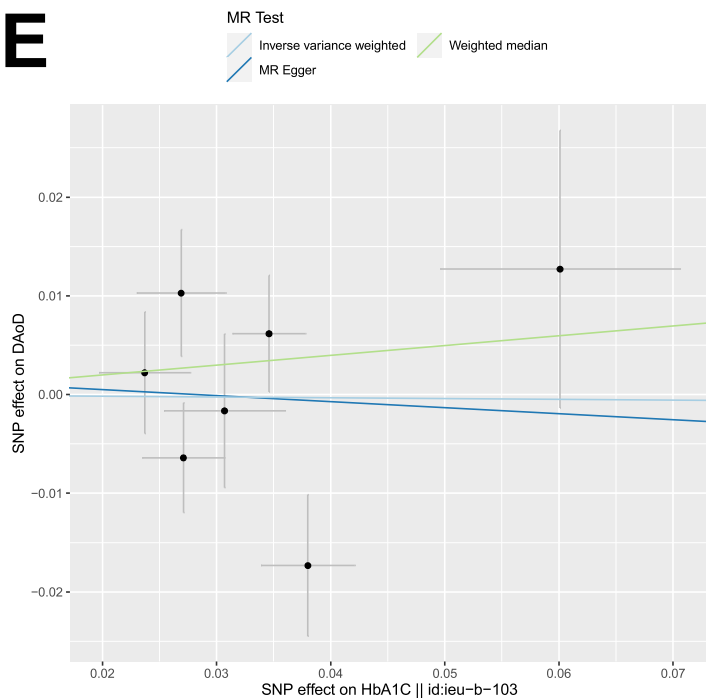**F**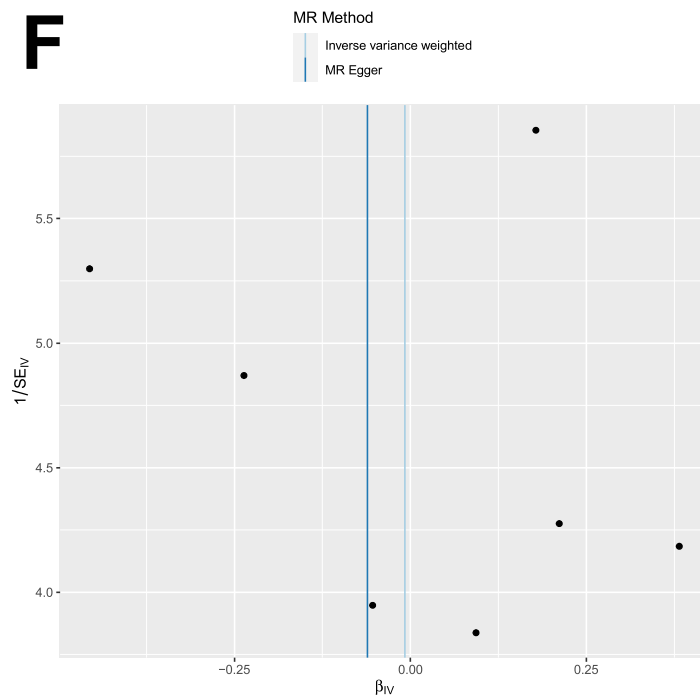

Supplement: Supplementary file 2 — Additional file 2: Figure S1. Scatter plots and funnel plots of MR analyses for HbA1c with TAA, AAoD and DAoD. Scatter plot (A) and funnel plot (B) of MR analysis for HbA1c with TAA. Scatter plot (C) and funnel plot (D) of MR analysis for HbA1c with AAoD. Scatter plot (E) and funnel plot (F) of MR analysis for HbA1c with DAoD. HbA1c, glycated hemoglobin; TAA, thoracic aortic aneurysm; AAoD, ascending aortic diameter; DAoD, descending aortic diameter. [file 13098_2023_1101_MOESM2_ESM.pdf]

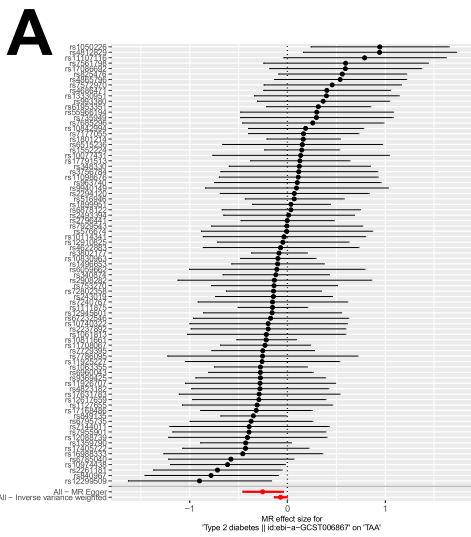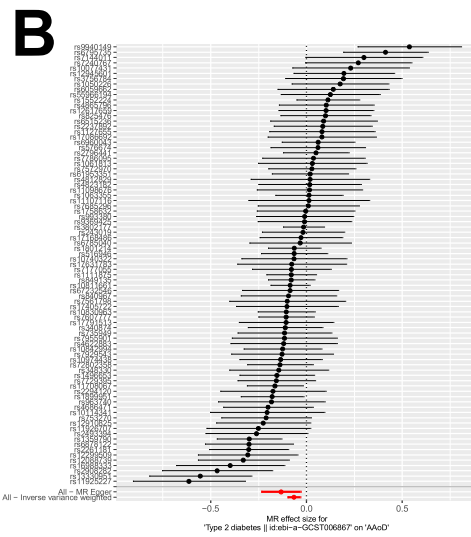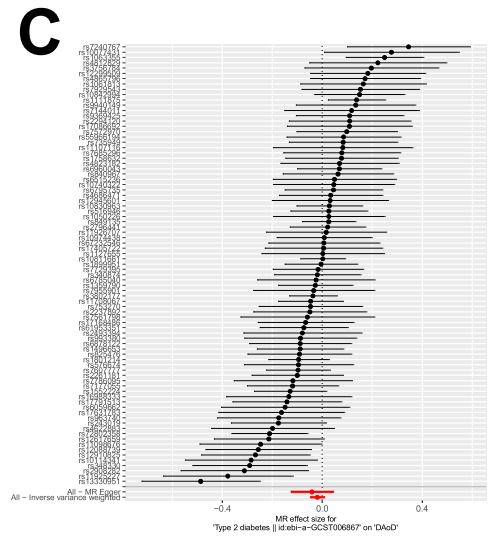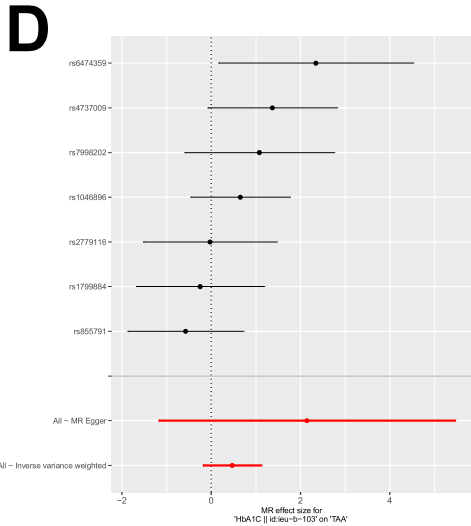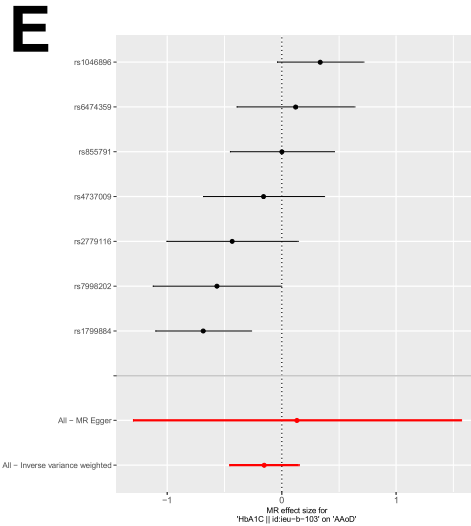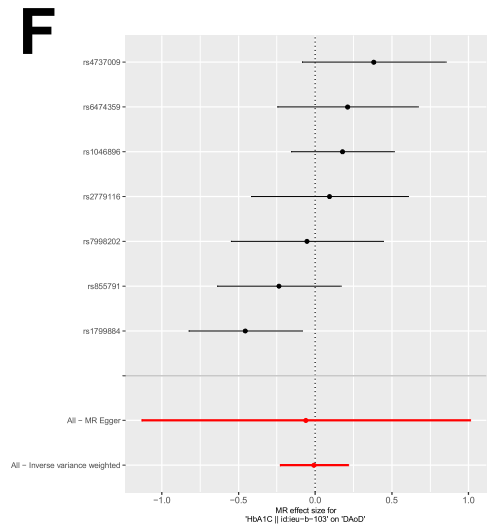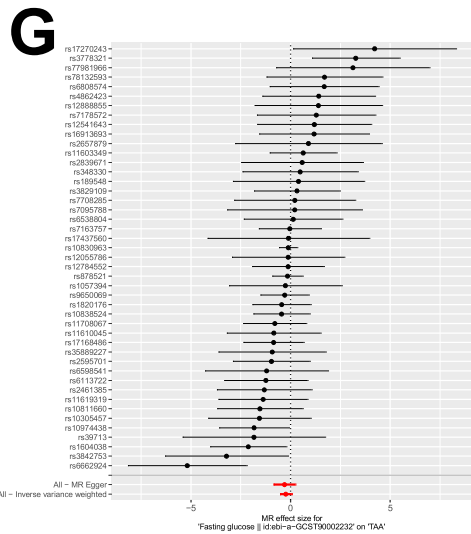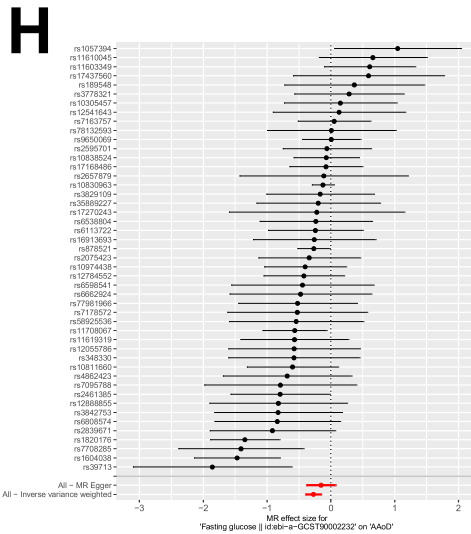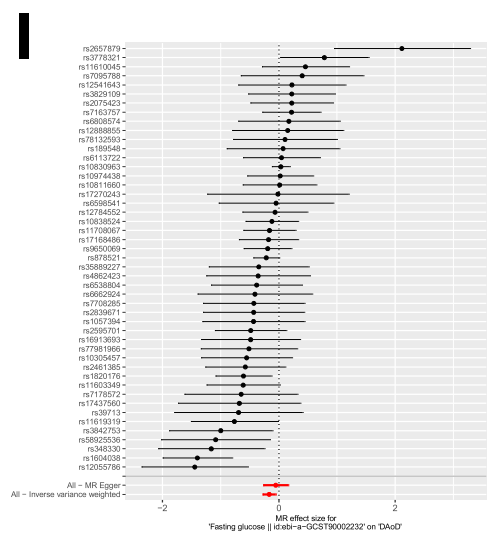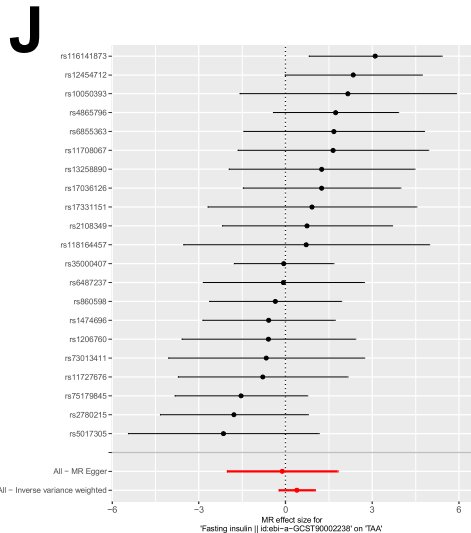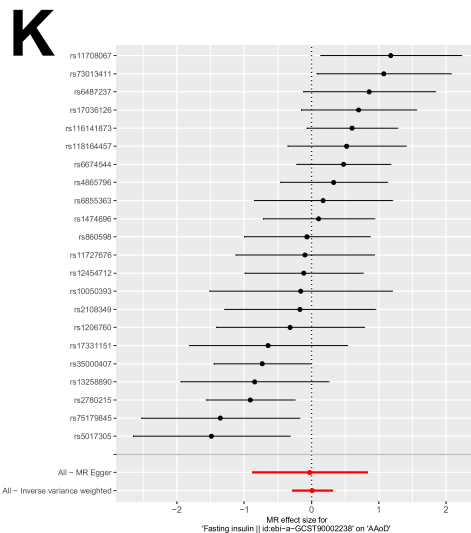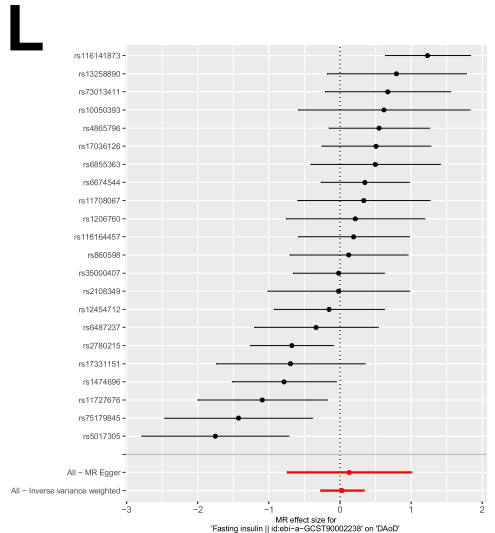

Supplement: Supplementary file 3 — Additional file 3: Figure S2. Forest plots of MR analyses for T2D with TAA (A), AAoD (B) and DAoD (C), for HbA1c with TAA (D), AAoD (E) and DAoD (F), for FG with TAA (G), AAoD (H) and DAoD (I), and for FI with TAA (J), AAoD (K) and DAoD (L). T2D, type 2 diabetes; HbA1c, glycated hemoglobin; FG, fasting glucose; FI, fasting insulin; TAA, thoracic aortic aneurysm; AAoD, ascending aortic diameter; DAoD, descending aortic diameter. [file 13098_2023_1101_MOESM3_ESM.pdf]

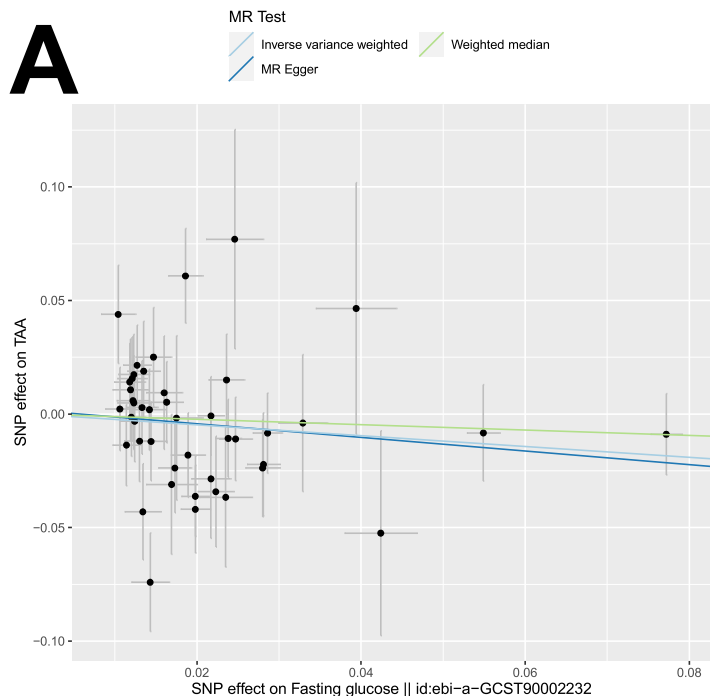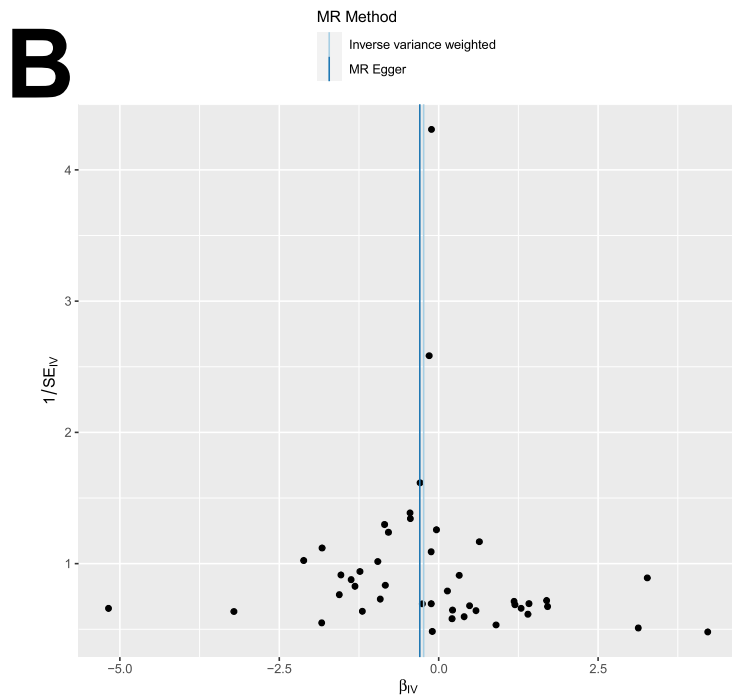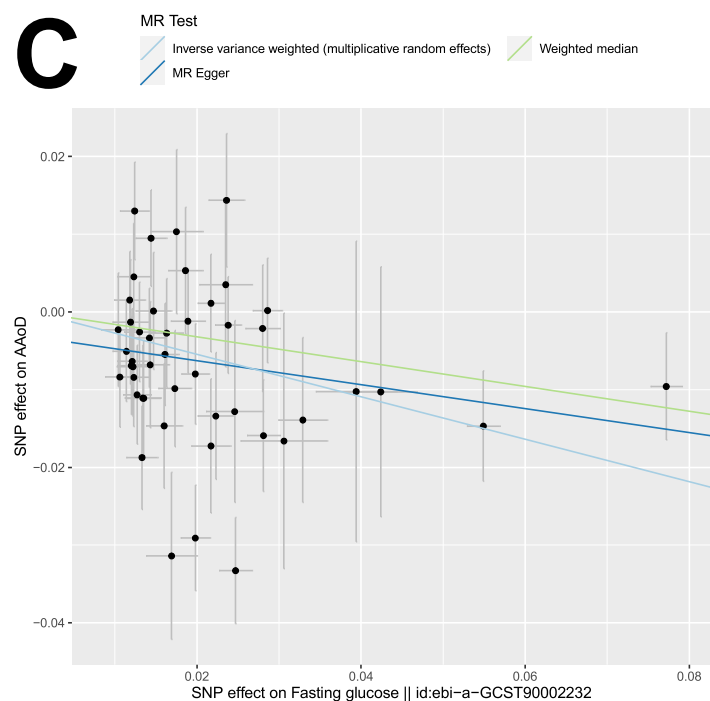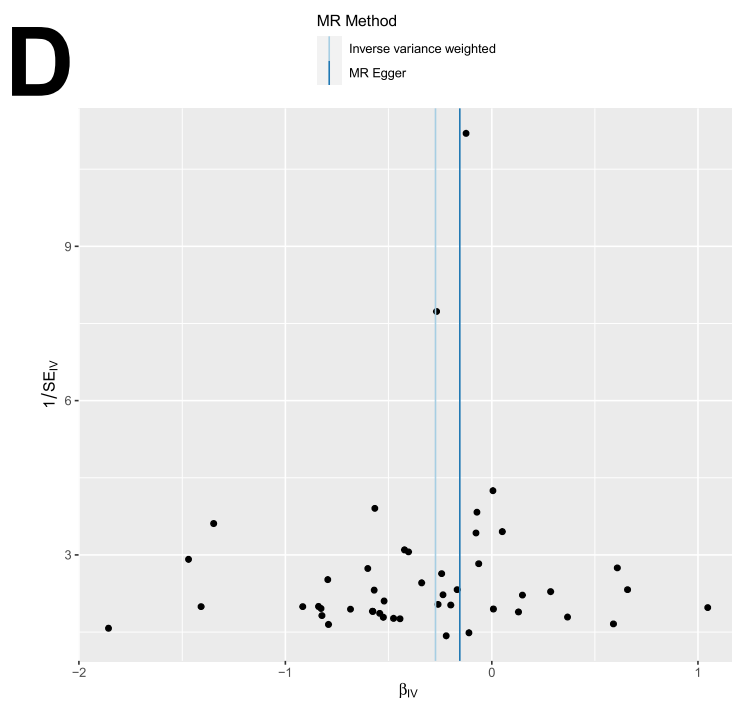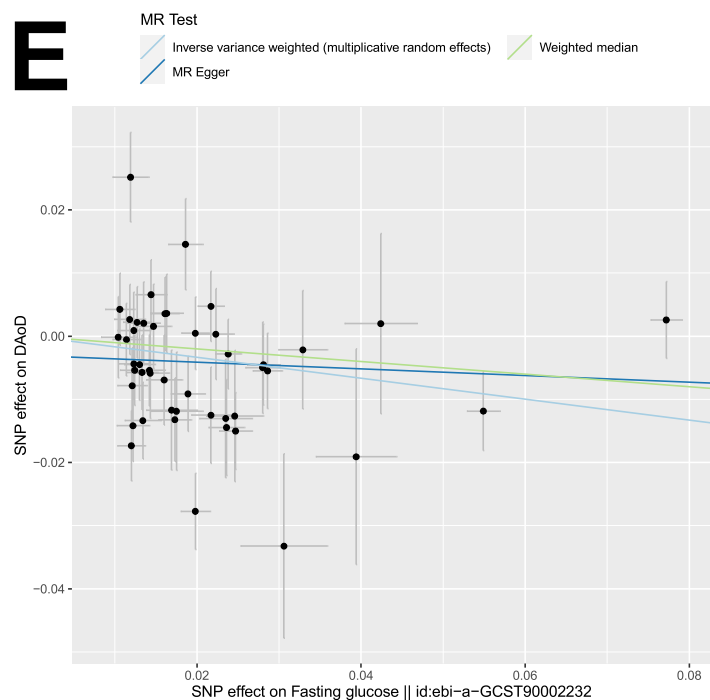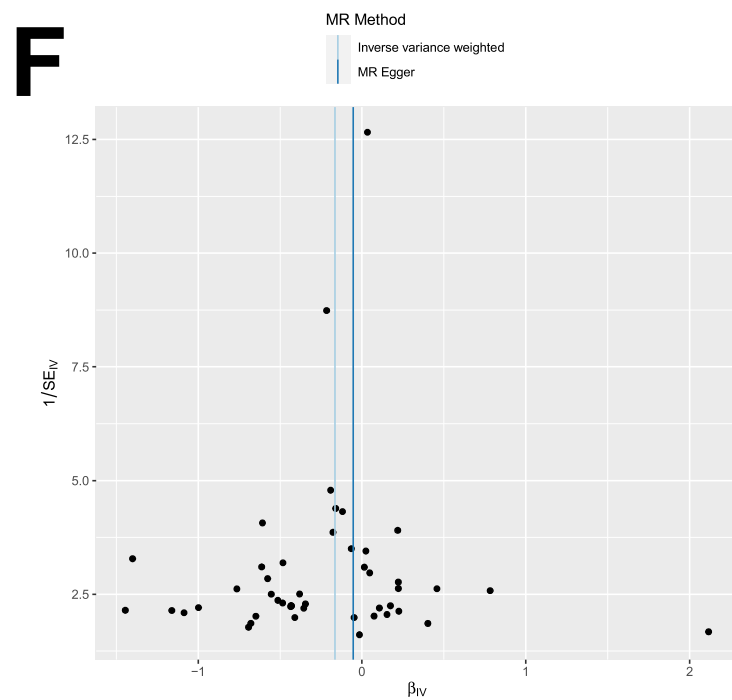

Supplement: Supplementary file 4 — Additional file 4: Figure S3. Scatter plots and funnel plots of MR analyses for FG with TAA, AAoD and DAoD. Scatter plot (A) and funnel plot (B) of MR analysis for FG with TAA. Scatter plot (C) and funnel plot (D) of MR analysis for FG with AAoD. Scatter plot (E) and funnel plot (F) of MR analysis for FG with DAoD. FG, fasting glucose; TAA, thoracic aortic aneurysm; AAoD, ascending aortic diameter; DAoD, descending aortic diameter. [file 13098_2023_1101_MOESM4_ESM.pdf]

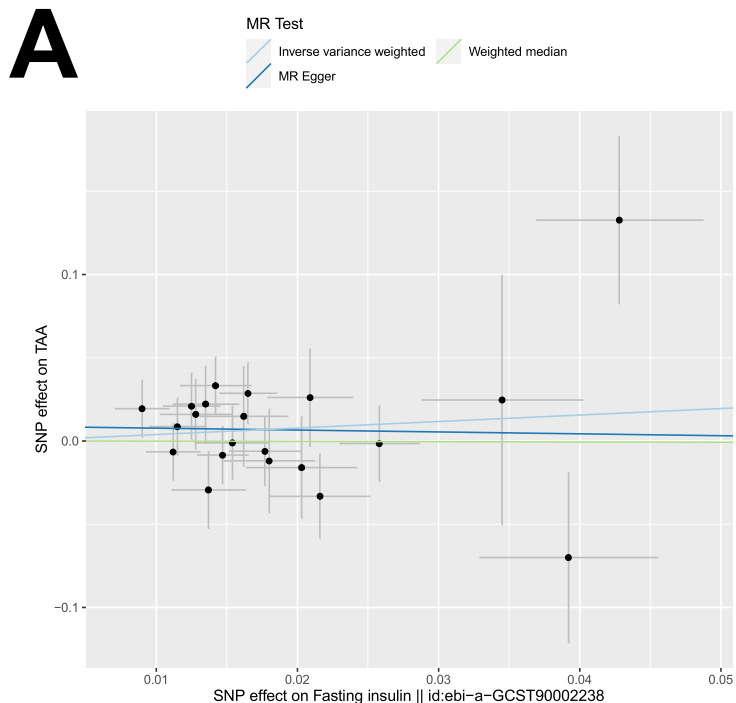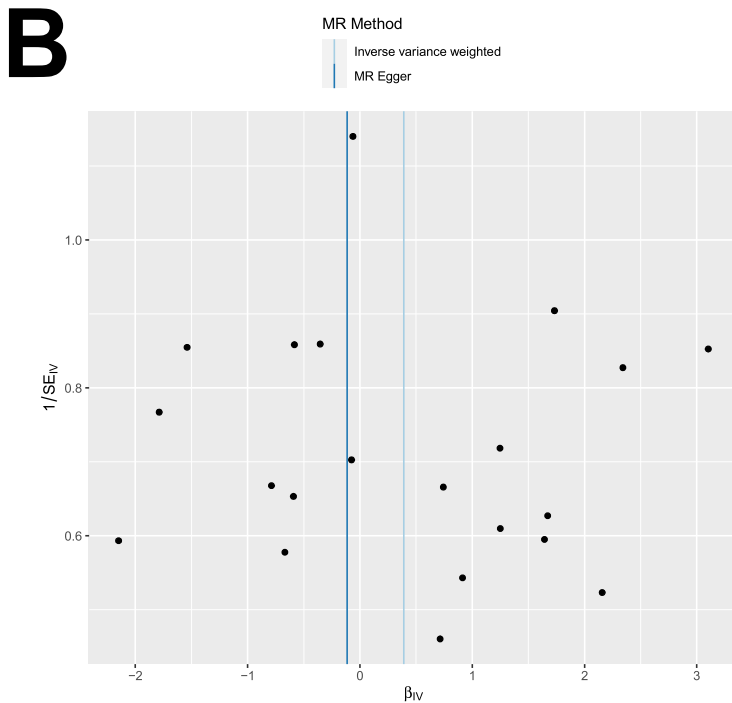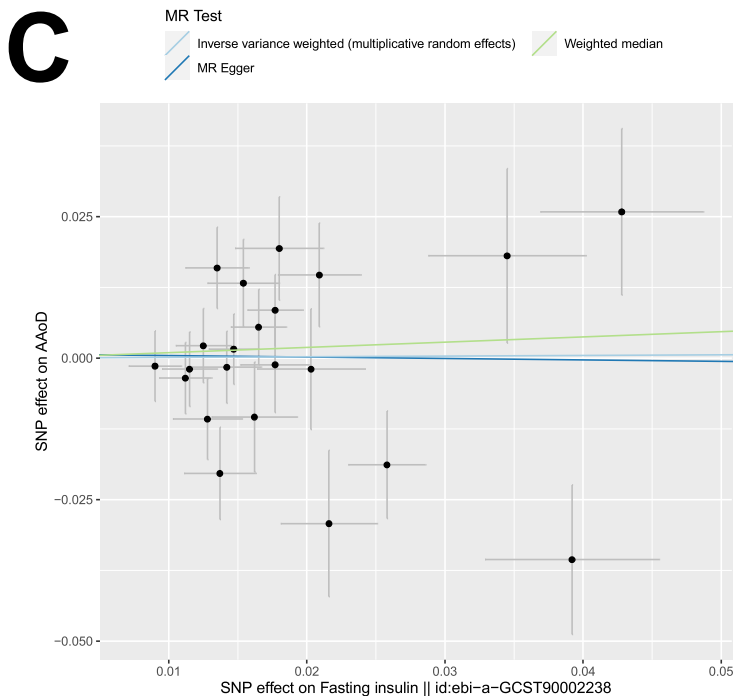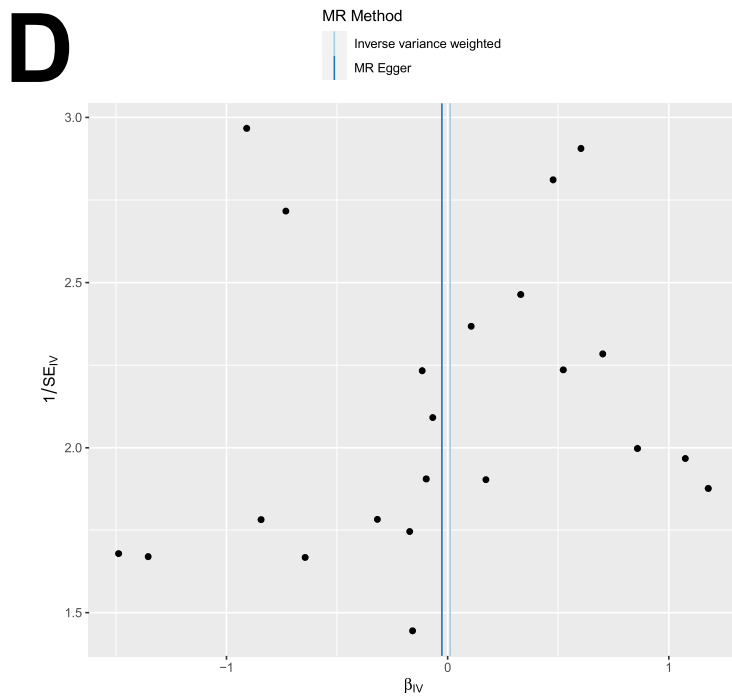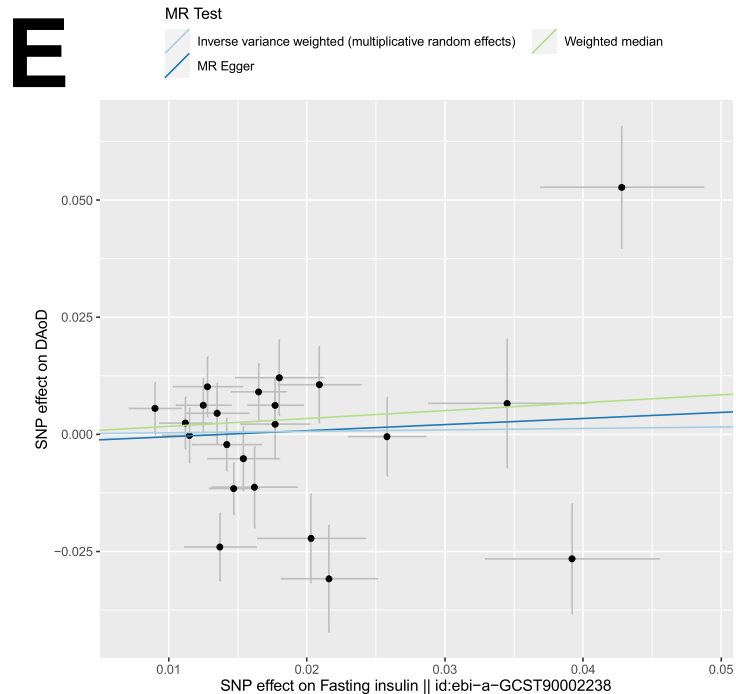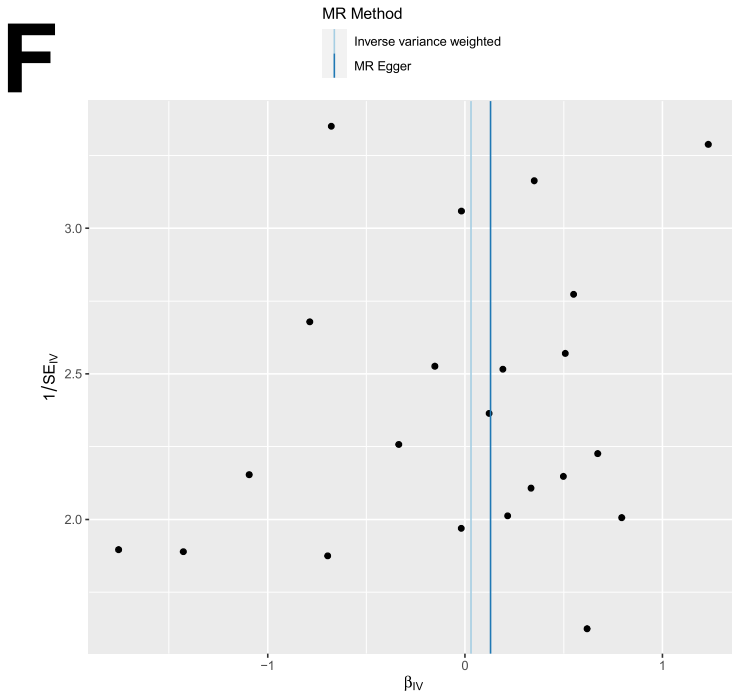

Supplement: Supplementary file 5 — Additional file 5: Figure S4. Scatter plots and funnel plots of MR analyses for FI with TAA, AAoD and DAoD. Scatter plot (A) and funnel plot (B) of MR analysis for FI with TAA. Scatter plot (C) and funnel plot (D) of MR analysis for FI with AAoD. Scatter plot (E) and funnel plot (F) of MR analysis for FI with DAoD. FI, fasting insulin; TAA, thoracic aortic aneurysm; AAoD, ascending aortic diameter; DAoD, descending aortic diameter. [file 13098_2023_1101_MOESM5_ESM.pdf]
